# A Carbon Nanofiber Electrochemical Sensor Made of FeMn@C for the Rapid Detection of Tert-Butyl Hydroquinone in Edible Oil

**DOI:** 10.3390/molecules30132725

**Published:** 2025-06-25

**Authors:** Yan Xiao, Yi Zhang, Zhigui He, Liwen Zhang, Tongfei Wang, Tingfan Tang, Jiaxing Chen, Hao Cheng

**Affiliations:** 1 GRG Metrology & Test (Nanning) Co., Ltd., Nanning 530000, China; xiaoyan@grgtest.com; 2 School of Biology, Food and Environment, Hefei University, Hefei 230601, China; yizhangkim@126.com (Y.Z.);; 3 Guangxi Engineering Research Center for Large-Scale Preparation & Nutrients and Hygiene of Guangxi Cuisine, Key Laboratory of Industrialized Processing and Safety of Guangxi Cuisine, Guilin Tourism University, Guilin 541006, China; hzg@gltu.edu.cn; 4 Guangxi Key Laboratory of Green Processing of Sugar Resources, Guangxi Liuzhou Luosifen Center of Technology Innovation, College of Biological and Chemical Engineering, Guangxi University of Science and Technology, Liuzhou 545006, Chinatangtingfan@163.com (T.T.)

**Keywords:** Prussian blue analogues, carbon nanofibers, differential pulse voltammetry, tert-butyl hydroquinone

## Abstract

Overuse of tert-butylhydroquinone (TBHQ) as a food antioxidant has the potential to pollute the environment and threaten human health. Therefore, it is imperative to develop precise and rapid methods to detect TBHQ in food products. In this study, Fe- and Mn-doped Prussian blue analogs (FeMn-PBAs) were prepared by co-precipitation, FeMn-PBAs/PAN was prepared by electrostatic spinning, and a novel FeMn@C/CNFs composite was prepared by carbonization in nitrogen. Bimetallic FeMn doping has been shown to reduce vacancy defects and enhance the structural stability of PBA. Furthermore, electrostatic spinning has been demonstrated to reduce the agglomeration of PBA nanoparticles, which are electrode-modifying materials with high stability and good electrical conductivity. The morphological and structural characteristics of the FeMn@C/CNF composites were examined using scanning electron microscopy (SEM), X-ray diffraction (XRD), transmission electron microscopy (TEM), and X-ray photoelectron spectroscopy (XPS). The electrochemical behavior of tert-butyl hydroquinone in FeMn@C/CNFs was studied by cyclic voltammetry (CV), differential pulse voltammetry (DPV), and chronocoulometry (CC). The results demonstrate that the sensor exhibits excellent repeatability, reproducibility, and anti-interference capabilities. The prepared electrochemical sensor can be effectively utilized for the detection of TBHQ in food samples such as soybean and peanut oil samples, proving its strong potential for practical applications.

## 1. Introduction

Tert-butylhydroquinone (TBHQ) is a food antioxidant commonly used to prevent food oxidation and spoilage. A significant advantage of TBHQ is that it does not alter the flavor of the materials to which it is added, thereby leading to its widespread use compared to other antioxidants [1,2,3]; However, the overuse of TBHQ can cause health problems in humans [4,5]. Numerous studies have shown that long-term and high-dose intake of TBHQ may increase the risk of cancer and that its metabolites are toxic. Moreover, TBHQ may affect T-cell function and suppress immune responses. Consequently, these effects can exacerbate allergic reactions and, in severe cases, become life threatening [6,7]. Currently, several methods can be used to accurately detect TBHQ, including high-performance liquid chromatography [8], gas chromatography [9], and spectrophotometry [10]. However, the sample pretreatment processes in these methods are cumbersome and result in longer detection times. In addition to the aforementioned approaches, electrochemical methods with the advantages of rapid detection, high sensitivity, and good reproducibility can be used to detect TBHQ [11]. The development of a more efficient and accurate TBHQ detection method could contribute to the maintenance of food quality and safety. Therefore, the preparation of a novel electrochemical sensor for the detection of trace TBHQ is essential.

Prussian blue (PB) and its analogs (PBA) are metal–organic skeletons (MOFs) consisting of nodes of transition metal ions linked by cyano ligands [12]. In recent years, high-quality PBAs with high crystallinity, few defects, and [Fe(CN)_6_]^3+/4+^ vacancies have demonstrated excellent coordination ability, high electrical conductivity, and high catalytic properties, which make them better electrode modification materials [13]. However, PBA is prone to phenomena such as lattice distortion or structural collapse during redox reactions and electrolyte erosion, which, in turn, leads to limited ion diffusion kinetics [14]. Meanwhile, researchers have found that PBA has a large gap for inserting large metal ions, and its three-dimensional network structure can avoid the structural changes caused by the insertion of metal ions [15,16], which, in turn, improves the stability of the sensor and enhances the ion transport efficiency and, hence, the performance of the sensor [17].

Examples of improving the performance of simple PBAs by synthesizing bimetals are now commonplace. For example, Tang et al. [18] reported a bimetallic Fe, Ni-doped PBA-filled hollow graphite tube (FeNiHCF@HGT) electrode for capacitive deionization. The synergistic effect of the transition metal iron, which readily transfers electrons and contributes to electrochemical reactivity, and manganese, which is stable and electrically conductive, improves the electrochemical properties of the material. Co-doping with iron and manganese in the PBA structure creates more active sites for electrochemical reactions, and the two metal ions can have a complementary effect, thus improving the electrocatalytic activity [19,20]. This plays a crucial role in maintaining the sensor’s reliability during repeated measurements and enhancing its sensitivity.

Despite the good structural stability of PBA after doping with Fe and Mn, agglomeration inevitably occurs during the synthesis process, mainly because of the fast crystallization rate of PBA during the synthesis process [21,22]. The agglomeration of PBA reduces the number of active sites, increases the electron transfer resistance, decreases the signal stability and reproducibility, slows down the response speed, and reduces the sensitivity [23]. This significantly limits the performance of the sensor. However, PBA aggregation was shown to be inhibited by the composite carbon network structure.

Among the various types of carbon-based materials, amorphous carbon nanofibers (CNFs) have the advantages of a highly controllable structure, high electrical conductivity, and stability, making them promising active support materials [24]. Electrospinning technologies have enabled the simple and rapid preparation of multifunctional CNFs. CNFs formed by electrospinning can be spontaneously crosslinked to form a 3D crosslinked network structure with a large specific surface area [25,26]. Incorporating CNFs into composite materials can enhance their electrical conductivity and stability, and the unique structure of the CNFs can ameliorate the agglomeration of PBA NPs. Therefore, PBA-doped CNFs serve as an effective means of preparing materials with excellent electrochemical performances.

In this study, Fe- and Mn-co-doped PBA materials were prepared using a co-precipitation method. Subsequently, FeMn@C/CNFs were prepared via electrospinning and carbonization. The prepared composite material was subjected to electrode modification to construct an electrochemical sensor for rapid detection of TBHQ. The constructed electrochemical sensor exhibited high stability, strong anti-interference ability, and a wide linear range for the detection of TBHQ. Accurate results were obtained by testing soybean oil and olive oil samples.

## 2. Experimental Section

### 2.1. Instruments and Reagents

The following instruments were used in this study: FEI Nova NanoSEM x30 ultra-high resolution field-emission scanning electron microscope (SEM; Thermo Fisher, Waltham, MA, USA), JEM 2100F field-emission transmission electron microscope (TEM; JEOL, Ltd., Tokyo, Japan); D8 ADVANCE Bruker X-ray diffractometer (XRD; Bruker, Karlsruhe, Germany); Thermo ESCALAB 250 Xi X-ray photoelectron spectrometer (XPS; Thermo Fisher, Waltham, MA, USA); CHI660E electrochemical workstation (CH Instruments, Shanghai, China); a three-electrode system consisting of a saturated Ag/AgCl electrode as the reference electrode; a Pt electrode as the auxiliary electrode; and a modified glassy carbon electrode (GCE, d = 3 mm) as the working electrode (Tianjin Lanlike Chemical Electronics High Technology Co., Ltd., Tianjin, China).

The following reagents were used: polyacrylonitrile (PAN, Mw = 50,000 g/mol), TBHQ (98%), N, N-dimethylformamide (DMF) (Aladdin Reagent Co. Ltd., Shanghai, China), manganese chloride (MnCl_2_), iron(III) chloride hexahydrate (FeCl_3_·6H_2_O), citric acid (Shanghai Macklin Biochemical Co., Ltd., Shanghai, China), ultrapure water, 0.1 M phosphate-buffered saline (PBS, with the pH adjusted using phosphoric acid and potassium hydroxide), and soybean oil and peanut oil (purchased from a Walmart store in Liuzhou, China). All the chemical reagents used in this study were of analytical grade, and no purification treatment was applied before use. Ultrapure water was used in all the experiments.

### 2.2. Synthesis of FeMn-PBAs

FeMn-PBAs were synthesized using a co-precipitation method [27]. To this end, 1.9 g of MnCl_2_, 1.3 g of FeCl_3_·6H_2_O, and 0.1 g of citric acid were dissolved in 20 mL of water to form solution A. Furthermore, 4.2 g of K_4_[Fe(CN)_6_]·3H_2_O and 0.1 g of citric acid were dissolved in 20 mL of water to form solution B. Subsequently, solution A was added to solution B, and the mixture was reacted at 60 °C for 5 min. At the end of the reaction, the reaction mixture was cooled to 25 °C; 10 mL of 0.5 M NaCl solution was added, and the mixture was mixed uniformly. Finally, centrifugation and washing with ultrapure water were performed ten times, and the FeMn-PBAs were obtained by drying at 60 °C for 12 h.

### 2.3. Preparation of Fe/Mn@C/CNFs

**Figure 1:**
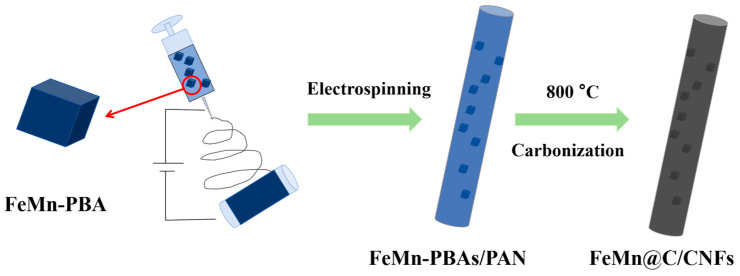
Flowchart of FeMn@C/CNFs produced by the electrostatic spinning method.

FeMn-PBA (0.6 g) was dissolved in 1.2 g of PAN in 10 mL of DMF solution. Stirring was performed in a 60 °C water bath to obtain the precursor solution. Subsequently, the precursor solution was loaded into an electrospinning syringe, and FeMn-PBA/CNFs were obtained by electrospinning. Finally, the FeMn-PBAs/CNFs were calcined at 800 °C in an N_2_ atmosphere for 2 h to obtain FeMn@C/CNFs. Figure 1 shows a schematic representation of the preparation protocol.

The reader is directed to the Supplementary Materials for a detailed examination of the specific parameters governing the electrostatic spinning process.

### 2.4. Preparation of FeMn@C/CNFs/GCE

The GCE was polished with α-Al_2_O_3_ (0.05 μm) powder and ultrasonically washed using ultrapure water and ethanol. FeMn@C/CNFs were added to a DMF solution to prepare a uniformly dispersed modification solution. Finally, a 5 μL drop of the modification solution was applied to the electrode surface. The electrode was dried naturally at 25 °C to obtain FeMn@C/CNFs/GCE.

### 2.5. Electrochemical Testing

The performance of the FeMn/CNF/GCE electrochemical sensor was investigated using differential pulses (DPV) and cyclic voltammetry (CV). DPV testing was performed using pulse periods, pulse amplitudes, pulse widths, and potential steps of 0.5, 50, 50, and 4 mV. CV was conducted using two sweep segments with a sample interval of 0.001 V. In CV, [Fe(CN)_6_]^3+/4+^ was used as the redox probe.

## 3. Results and Discussion

### 3.1. Material Characterization

**Figure 2:**
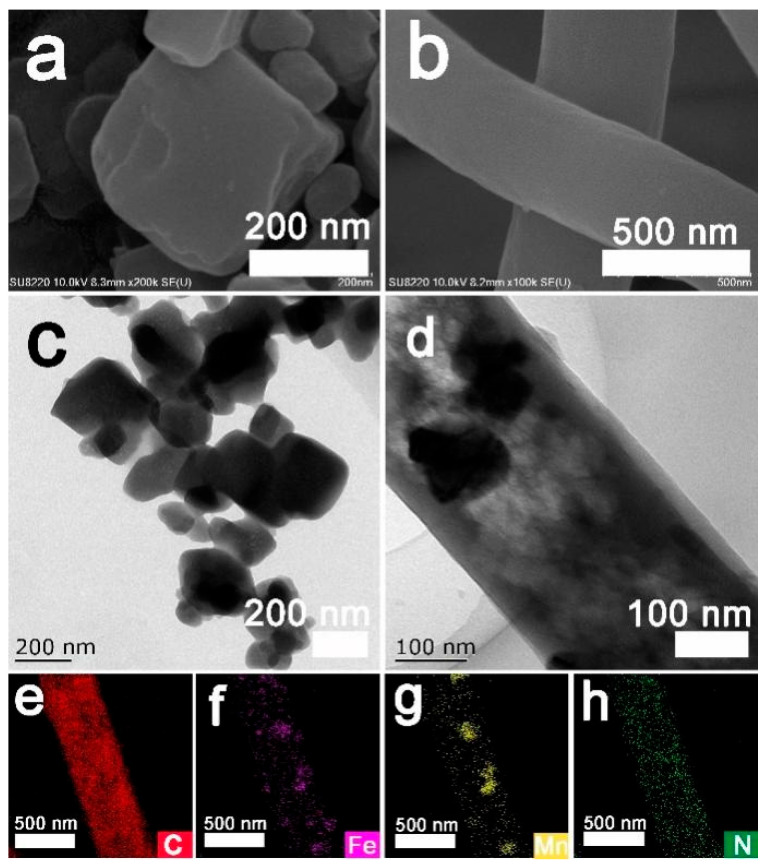
(**a**) SEM map of FeMn-PBAs; (**b**) SEM map of FeMn@C/CNFs; (**c**) TEM map of FeMn-PBAs; (**d**) TEM map of FeMn@C/CNFs; (**e**–**h**) maps of C, Fe, Mn, and N of FeMn@C/CNFs.

The morphologies of the prepared FeMn-PBAs and FeMn@C/CNFs were investigated using SEM and TEM. As shown in the SEM image of the FeMn-PBAs in Figure 2a, the prepared FeMn-PBAs exhibited a cubic morphology [28]. Figure 2b shows an SEM image of the FeMn@C/CNFs, wherein cylindrical fibers of uniform thickness can be observed. The SEM characterization of FeMn@C/CNFs at varying scales is shown in Figure S1. The TEM image of the FeMn-PBAs in Figure 2c confirms their cubic morphology. The TEM image of FeMn@C/CNFs in Figure 2d shows that FeMn-PBAs were present in the CNFs, even after heat treatment in N_2_. Figure 2e–h show the distributions of C, Fe, Mn, O, and N in the fibers. (The distribution of O is illustrated in Figure S2 in the Material Characterization) The contents of C, N, O, Fe, and Mn in CNFs were 84.6 wt%, 2.3 wt%, 6.9 wt%, 4.6 wt%, and 2.3 wt%, respectively, as scanned by EDS energy spectroscopy, shown in Figure S3.

**Figure 3:**
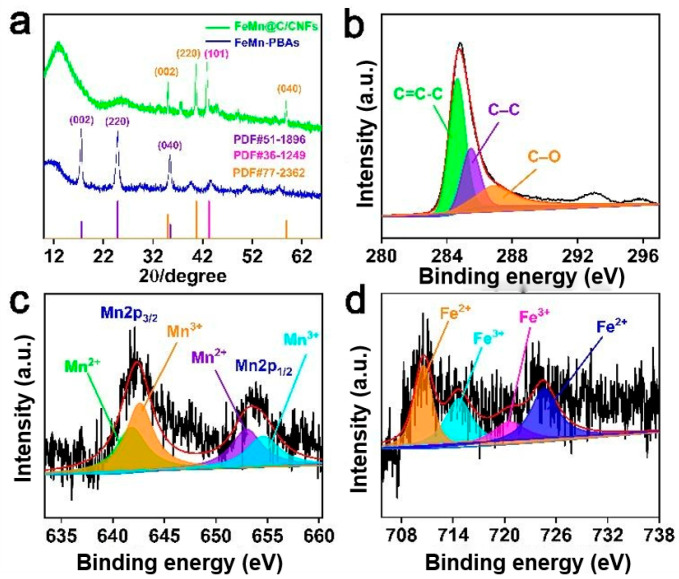
(**a**) XRD plot of FeMn-PBAs and FeMn@C/CNFs. XPS profiles of (**b**) C1s; (**c**) Mn2p; and (**d**) Fe2p of FeMn@C/CNFs.

Figure 3a presents the XRD patterns of the prepared FeMn-PBAs and FeMn@C/CNFs. The characteristic peaks of the FeMn-PBA particles were located at 2θ = 15.5°, 24.9°, and 35.5°, which correspond to the (002), (220), and (040) crystal planes, respectively. These findings are consistent with the powder diffraction file card (PDF) #51-1896, which demonstrates that FeMn-PBA particles were successfully prepared [29]. The characteristic peak of the FeMn@C/CNFs composite material at 43.2° is attributed to the (101) crystal plane of Fe_2_C (PDF #51-1896). The characteristic peaks occurring at 2θ = 35.0°, 40.7°, and 58.9°, respectively, corresponded to the (111), (200), and (220) crystal planes of (FeO)_0.099_(MnO)_0.901_ (PDF #77-2362).

The composition of the FeMn@C/CNFs composite was characterized using XPS. In the high-resolution XPS profile of C1s shown in Figure 3b, the three peaks correspond to C=C-C (284.6 eV), C-C (285.5 eV), and C-O (286.9 eV) [30,31]. The high-resolution profile of Mn2p in Figure 3c was split into two spin orbitals: Mn2p_1/2_ (653.4 eV) and Mn2p_3/2_ (642.4 eV). There are two types of peaks for Mn2p, namely Mn^2+^ (641.8 and 653.0 eV) and Mn^3+^ (642.6 and 654.6 eV) [32,33]. The high-resolution XPS profile of Fe2p in Figure 3d is attributed to Fe^2+^ at 710.6 and 724.6 eV and Fe^3+^ (714.7 and 720.5 eV) [34].

### 3.2. Optimization of Electrochemical Conditions

**Figure 4:**
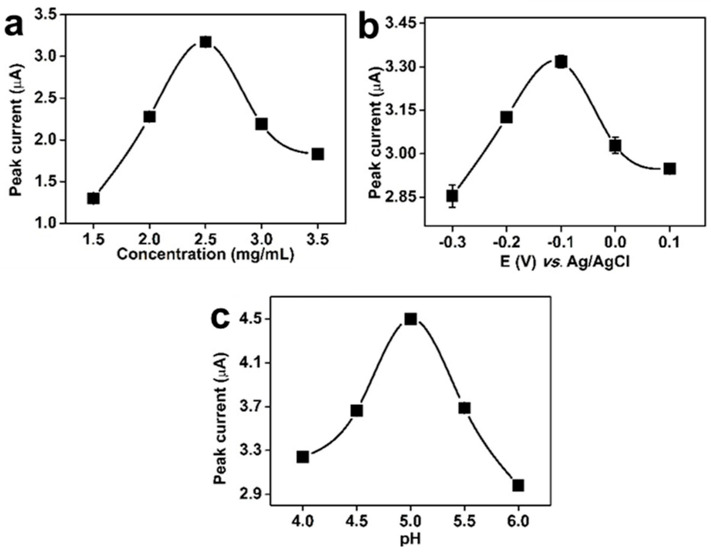
(**a**) Modification concentration of the FeMn@C/CNF material (pH = 7, E(v) = 0.00 V); (**b**) enrichment potential (concentration = 2.5 mg/mL, pH = 7); (**c**) different pH detection effects of the 5 μM tert−butyl hydroquinone (concentration = 2.5 mg/mL, E(v) = −0.10 V) (*n* = 3).

The influences of modification solution concentration, enrichment potential, and pH on the detection of 5 μM TBHQ by FeMn@C/CNFs/GCE were investigated by DPV by fixing the volume of the modification solution for the FeMn@C/CNFs composite material at 5 μL. Figure 4a shows the changes in the peak current when the modified solution concentration was within the range of 1.5–3.5 mg/mL. The peak current for TBHQ detection by the FeMn@C/CNFs/GCE increased gradually with an increasing modification solution concentration and reached a maximum value at a concentration of 2.5 mg/mL. This may be because the number of active sites on the electrode surface increases as the amount of electrode surface modification material increases. These active sites promote redox reactions. Further increases in the modification solution concentration led to a decrease in the peak current. This phenomenon may be attributed to the gradual thickening of the modifier layer on the electrode surface, which subsequently impedes electron transfer. Therefore, a modification solution concentration of 2.5 mg/mL was selected as the optimum concentration for further experiments.

Figure 4b shows the effect of the enrichment potential within the range of −0.3–0.1 V on the peak current. The peak current gradually increased and reached the maximum value when the enrichment potential was increased from −0.3 to −0.1 V. Further increases in the enrichment potential led to a decrease in peak current. This phenomenon can be attributed to the fact that within the pH 5.0 PBS buffer, the peak-out position of TBHQ falls within the range of −0.1 and 0.1 V. Consequently, when the enrichment voltage exceeds the response potential of TBHQ, alternative side reactions become predominant. This, in turn, resulted in a rapid decline in the current signal, thereby hindering the detection of TBHQ. Therefore, −0.1 V was selected as the optimal enrichment potential.

Figure 4c shows the effect of the pH within the range of 4.0–6.0 on the peak current. With an increase in the pH from 4.0 to 5.0, the peak current increased gradually and reached a maximum at pH 5.0. When the pH was increased from 5.0 to 6.0, the peak current exhibited a decreasing trend, which can be attributed to the increase in the concentration of hydroxyl anions in the solution concomitant with the increase in pH. The hydroxyl anions occupied the active sites that would otherwise be occupied by TBQH, thereby resulting in a decrease in the current signal. This indicates that the optimal pH was 5.0.

### 3.3. Influence of pH and Scan Rate

**Figure 5:**
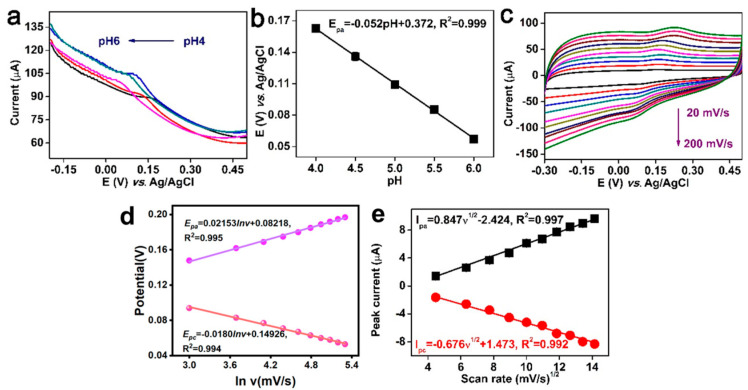
(**a**) DPV plot of tert−butyl hydroquinone at 5 μM detected by FeMn@C/CNFs/GCE under different pH conditions; (**b**) linear plot of different pH and oxidation peak potential (E_pa_); (**c**) CV plot in 5 μM TBHQ at different sweeps; (**d**) linear fit curve of the logarithm of the scan rate; (**e**) CV curves of peak current (I_p_) at different sweep rates versus the square root of the sweep rate (ν^1/2^) are linear (*n* = 3).

Figure 5a shows the DPV profiles of FeMn@C/CNFs/GCE for detecting 5 μM TBHQ under different pH conditions. The oxidation peak potential gradually shifted in the negative direction as the pH increased from 4.0 to 6.0. The pH exhibited a good linear relationship with the oxidation peak potential (E_pa_), with the linear regression equation being E_pa_ = −0.052 pH + 0.372 (R^2^ = 0.999) (Figure 5b). The slope of the linear equation was 52 mV/pH, which is close to the theoretical slope of Nernst’s equation. This implied that the number of proton transfers was equal to the number of electron transfers in the electrochemical reaction of TBHQ [35].

To investigate the influence of the scan rate on the performance of the FeMn @C/CNFs/GCE, cyclic voltammetry (CV) tests were conducted over a scan rate range of 20–200 mV/s. Figure 5c illustrates the CV curves of 5 μM TBHQ at different scan rates, where a gradual upward shift in the anodic peak potential (E_pa_) was observed with increasing scan rates. There is a linear relationship between the peak potential (E_p_) and the logarithm of the scanning rate (lnν) in Figure 5d. The Laviron Equations (1) and (2) were employed to calculate the electron transfer number (n) and transfer coefficient (β) of the analyte, providing insights into the electrochemical reaction kinetics [36,37].

(1) $$E_{pa}=E_{0}+\frac{RT}{\beta nF}-\frac{RT}{\beta nF}ln\nu$$

(2) $$E_{pa}=E_{0}+\frac{RT}{(1-\beta )nF}-\frac{RT}{(1-\beta )nF}ln\nu$$

**Figure 6:**
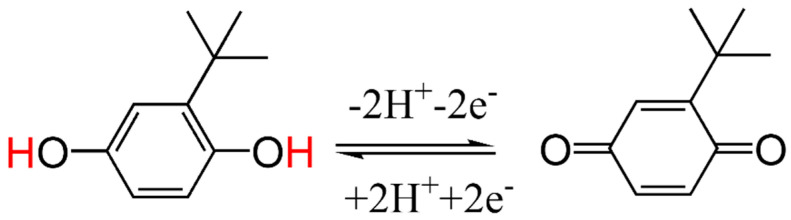
Schematic of the redox mechanism of tert−butyll hydroquinone on FeMn@C/CNFs/GCE.

In the formula, R is the gas constant (8.314 J·mol^−1^·K^−1^), T is the temperature in Kelvin (K), F is the Faraday constant (96,485 C·mol^−1^), ν is the scanning rate (mV/s), and β is the transfer coefficient. As presented in Figure 5d, the transfer coefficient (β) was obtained through linear regression of the cathodic potential (E_pc_) or anodic potential (E_pa_) against the natural logarithm of the scan rate. This analysis yielded calculated values of 0.7 for β and 2.037 for n (Figure 6). These findings suggest that the oxidation involves a two-electron transfer process.

The relationships between the scan rates and TBHQ oxidation and reduction peak currents were investigated using the CV profiles. Figure 5c shows that within the scan rate range of 20–200 mV s^−1^, the oxidation peak current (I_pa_) and reduction peak current (I_pc_) increased gradually with an increase in the scan rate. Both I_pa_ and I_pc_ exhibited significant linear relationships with the square root of the scan rate (ν^1/2^), which could be expressed as I_pa_ (μA) = 0.847 ν^1/2^(mV^1/2^ s^−1/2^) − 2.424 (R^2^ = 0.997) and I_pc_ (μA) = −0.676 ν^1/2^(mV^1/2^ s^−1/2^) + 1.473 (R^2^ = 0.992). These results demonstrated that the reaction of TBHQ with FeMn@C/CNFs/GCE was diffusion controlled [38].

### 3.4. Electrochemical Behaviors of Different Electrodes

**Figure 7:**
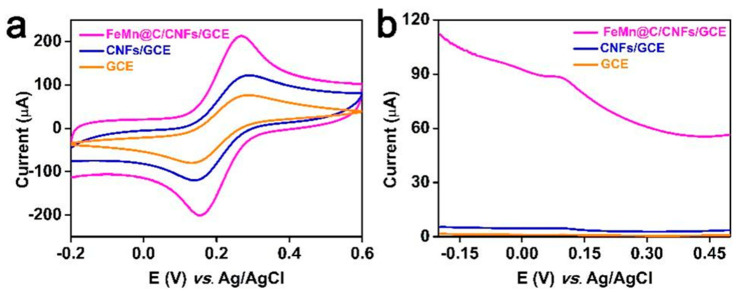
CV comparison of FeMn@C/CNFs/GCE, CNFs/GCE, and GCE (**a**) in [Fe(CN)_6_]^3+/4+^ and 0.1 M KCl containing 5 mM; (**b**) DPV detection of 5 μM tert−butyl hydroquinone.

Figure 7a compares the electrochemical performances of FeMn@C/CNF/GCE, CNF/GCE, and GCE by CV. The oxidation and reduction peak currents of FeMn@C/CNF/GCE were significantly higher than those of CNF/GCE and GCE. The DPV profiles of the different electrodes in Figure 7b indicate that FeMn@C/CNF/GCE had higher oxidation peak currents than CNF/GCE and GCE. This demonstrates the superiority of FeMn@C/CNFs over the CNFs.

To assess the conductivity of the modified electrodes, the electrochemically active surface area (ECSA) was evaluated using the Randles–Sevcik equation: [39].

(3) $$I_{pa}=2.69\times 10^{5}\times A\times n^{1/2}\times D^{1/2}\times C\times \nu^{1/2}$$

The equation employs I_pa_, n, A, C, D, and ν to denote the anodic peak current, electron transfer number, electrochemically active surface area, concentration of the electroactive species, diffusion coefficient (7.6 × 10^6^ cm^2^/s), and scan rate, respectively. The specific surface areas of the bare GCE, CNF/GCE, and FeMn@C/CNF/GCE were 0.009, 0.018, and 0.058 cm^2^, respectively. Among the electrodes tested, the FeMn@C/CNFs/GCE demonstrated the highest electrochemically active specific surface area. This enhanced surface area not only facilitates electron transfer kinetics but also significantly improves the analytical performance of the sensor.

### 3.5. Establishment of Standard Curves

**Figure 8:**
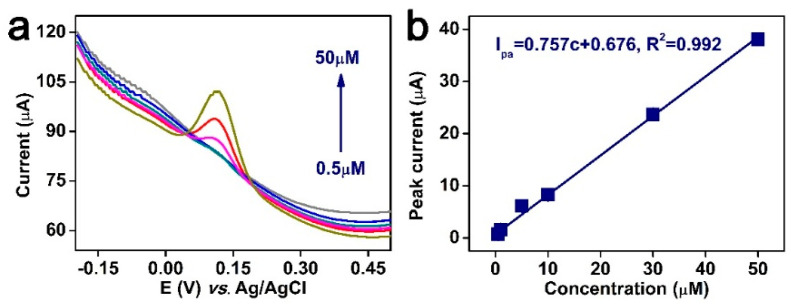
(**a**) DPV plot of tert−butyl hydroquinone at different concentrations by FeMn@C/CNFs/GCE and (**b**) linear plot between tert−butyl hydroquinone and peak current (*n* = 3).

Under the optimum test conditions, TBHQ at different concentrations was detected using FeMn@C/CNFs/GCE to investigate the electrical signal patterns. Figure 8a shows the DPV profiles within the concentration range of 0.5–50 µM. The oxidation peak current gradually increased and exhibited a good linear relationship with an increasing TBHQ concentration. Figure 8b shows that the linear equation of the relationship between oxidation peak current and TBHQ concentration is I_pa_ = 0.757c + 0.676 (R^2^ = 0.992), with a limit of detection (LOD) of 0.041 μM (S/N = 3).

As shown in Table 1, the performance of the FeMn@C/CNF-based TBHQ electrochemical sensor was compared with that of other sensors reported in the literature. Although some of the sensors exhibit higher sensitivity in the detection limit and linear range, the present sensor offers advantages in simplicity due to the synergistic design of the FeMn bimetallic active sites and 3D CNFs structures. The sensor excels in terms of cost control and simplicity, providing a practical technical solution for the rapid and low-cost detection of TBHQ.

### 3.6. Assessment of Repeatability and Reproducibility

**Figure 9:**
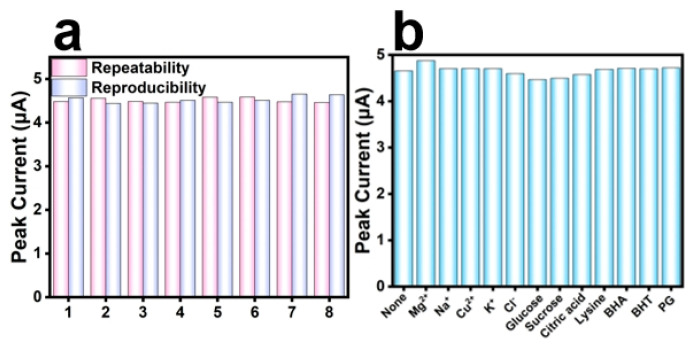
(**a**) Repeatability and reproducibility of 5 μM tert-butylhydroquinone detected by FeMn@C/CNFs/GCE; (**b**) anti-interference capability of FeMn@C/CNFs/GCE.

The reproducibility and repeatability of electrochemical detection of 10 μM TBHQ under optimum test conditions were investigated using FeMn@C/CNFs/GCE electrodes. As shown in Figure 9a, repeatability was assessed using a single FeMn@C/CNFs/GCE electrode for eight replicate measurements of 5 μM TBHQ. Further, reproducibility was determined using eight FeMn@C/CNFs/GCE electrodes from the same batch for detecting 5 μM TBHQ, with each electrode used once. The relative standard deviations for the repeatability and reproducibility were 1.29 and 1.83%, respectively. These results confirmed that FeMn@C/CNF/GCE possessed good reproducibility and repeatability.

In the buffer solution containing 5 μm tert-butylhydroquinone, 300-fold excess of Na^+^, Mg^2+^, Cu^2+^, and K^+^ and 100 times glucose, sucrose, and citric acid were added, respectively. The electrical signals of 20 times L-lysine, butylhydroxyanisole (BHA), butylhydroxytoluene (BHT), and propyl gallic acid (PG) were compared with those of the detection of tert-butylhydroquinone without interference substances. The results are shown in Figure 9b. The peak current changes were less than 10%, and the presence of interfering substances had no significant effect on the detection of tert-butylhydroquinone. Thus, this material has potential for practical applications.

### 3.7. Testing Actual Samples

The prepared FeMn@C/CNF/GCE sensor was used to detect TBHQ in soybean and peanut oils, and its performance in practical applications was evaluated. Here, 1 mL of soybean oil was mixed with 5 mL of methanol, and the mixture was ultrasonically treated for 30 min. The supernatant was collected by centrifugation, and this process was repeated three times to ensure better purification of the soybean oil. Finally, the methanol was diluted to a volume of 100 mL in a volumetric flask. Peanut oil was processed using the same method. Using a 10 mL pipette, 10 mL of the mixed solution was transferred from a 100 mL volumetric flask to a 10 mL beaker. The recovery rates of TBHQ from the soybean and peanut oils were determined using a standard addition method.

As shown in Table 2, the recovery rates of the soybean and peanut oils were 99.330–102.623% and 95.540–99.900%, respectively. According to the Codex Alimentarius Commission (CAC), the theoretical TBHQ concentration exceeding the permissible limit is 6.43 μM in soybean oil and 6.39 μM in peanut oil. These concentrations fall within the linear range and well above the detection limit. However, TBHQ was not detected in the actual oil samples. Therefore, spiking experiments were conducted, and the recovery results confirmed that the sensor is effective for detecting TBHQ in re-formaldehyde samples.

## 4. Conclusions

A FeMn@C/CNFs composite material for detecting TBHQ was prepared by electrospinning. The morphological structure of the FeMn@C/CNFs composite material was determined using a series of characterization methods, and its electrochemical performance was assessed. Our results indicated that the prepared FeMn@C/CNF/GCE sensor possessed superior repeatability, reproducibility, and anti-interference ability. Within the TBHQ concentration range of 0.5–50 µM, the TBHQ concentration and peak current exhibited good linear relationships, represented by the linear equation I_pa_ = 0.757c + 0.676 (R^2^ = 0.992), with the LOD being 0.041 μM. Accurate results were obtained when the prepared FeMn@C/CNF/GCE sensor was used to detect TBHQ in soybean oil and peanut oil samples. This confirmed that the FeMn@C/CNF/GCE sensor has immense potential for a wide range of practical applications.

## Figures and Tables

**Table 1:** Comparison of FeMn@C/CNFs with other reported works in TBHQ detection.

Modified Electrode	LOD (μM)	Linear Range (μM)	References
AuNPs/GCE	0.48	1.2–16.8	[40]
SPE-MWCNT	0.34	0.5–10	[41]
Peroxidase/Naf/SEP/CNT/CPE	2.47	9.9–59.1	[42]
ZnO TPHS@GO/GCE	0.02	0.13–10.83	[43]
MWCNT/CPE	0.487	1–10	[44]
MIP/AgNP/POM/rGO/GCE	0.0000148	0.0005–0.0015	[45]
ZnCuMg TMO/β-CD-CB/SPCE	0.001	0.031–12.56	[46]
FeMn@C/CNFs	0.041	0.5–50	This work

**Table 2:** FeMn@C/CNFs/GCE in soybean and olive oil samples (*n* = 3).

Sample	Added/(μmol/L)	Found ± SD/(μmol/L)	Recovery/%
Soybean oil	0.000	–	–
	3.000	3.073 ± 0.081	102.433
	20.000	19.886 ± 0.436	99.330
	40.000	41.049 ± 0.383	102.623
Peanut oil	0.000	–	–
	1.000	0.954 ± 0.030	95.540
	15.000	14.985 ± 0.368	99.900
	30.000	29.583 ± 1.039	98.610

## Data Availability

Data are contained within the article and Supplementary Materials.

## Supplementary Materials

The following supporting information can be downloaded at: https://www.mdpi.com/article/10.3390/molecules30132725/s1, TEXT S1. Specific parameters of the electrostatic spinning process; Figure S1: SEM characterization of FeMn@C/CNFs at varying scales; Figure S2: Mapping map of O of FeMn@C/CNFs; Figure S3: Characterization of the elemental content of FeMn@C/CNFs by EDS spectroscopy; Figure S4: (a) Contact angle of bare electrode; (b) Contact angle of FeMn@C/CNFs 33 material; Figure S5: Comparison of CVs for different electrodes without [Fe(CN)_6_]^3+/4+^; Figure S6: Impedance plots for different electrodes; Figure S7: Comparison of materials at different calcination temperatures.

## Abbreviations

The following abbreviations are used in this manuscript:

TBHQ	Tert-butylhydroquinone
PBAs	Prussian blue analogues
PB	Prussian blue
NPs	Nanoparticles
CNFs	Carbon nanofibers
SEM	Scanning electron microscope
TEM	Transmission electron microscope
XRD	X-ray diffractometer
XPS	X-ray photoelectron spectrometer
GCE	Glassy carbon electrode
PAN	Polyacrylonitrile
DMF	N, N-dimethylformamide
PBS	Phosphate-buffered saline
DPV	Differential pulse voltammetry
CV	Cyclic voltammetry
CC	Chronocoulometry
PDF	Powder diffraction file
