# Supplementary material for: A Carbon Nanofiber Electrochemical Sensor Made of FeMn@C for the Rapid Detection of Tert-Butyl Hydroquinone in Edible Oil
Source: Molecules. 2025 Jun 25;30(13):2725. doi: 10.3390/molecules30132725 (PMC12251489; doi:10.3390/molecules30132725)
Supplement: Supplementary file 1 [file molecules-30-02725-s001.zip › molecules-3641776-supplementary.pdf]

Supporting Information

A carbon nanofiber electrochemical sensor made of FeMn@C for rapid detection of tert-butyl hydroquinone in edible oil

Xiao Yan[1], Zhang Yi[2], He Zhigui[3], Zhang Liwen[4], Wang Tongfei[4], Tang Tingfan[4], Chen Jiaxing[2], Cheng Hao*,[4]

1 GRG Metrology & Test (Nanning) Co., Ltd.

2 School of Biology, Food and Environment, Hefei University, Hefei 230601, China;

3 Guangxi Engineering Research Center for Large-Scale Preparation & Nutrients and Hygiene of Guangxi Cuisine, Key Laboratory of Industrialized Processing and Safety of Guangxi Cuisine, Guilin Tourism University, Guilin, 541006, Guangxi, China;

4 Guangxi Key Laboratory of Green Processing of Sugar Resources, Guangxi Liuzhou Luosifen Center of Technology Innovation, College of Biological and Chemical Engineering, Guangxi University of Science and Technology, Liuzhou 545006, Guangxi, China

* Correspondence: chenghao@gxust.edu.cn;

TEXT S1. Specific parameters of the electrostatic spinning process

To obtain the optimal fibre morphology, the high voltage of electrostatic spinning was set to 17 kV, the rotational speed of the roller to 180 r/min, the injection speed was 1.2 mL/h, and the distance between the tip of the injector and the spinning axis to 10 cm. Under these conditions, continuous and uniform fibre materials were obtained.

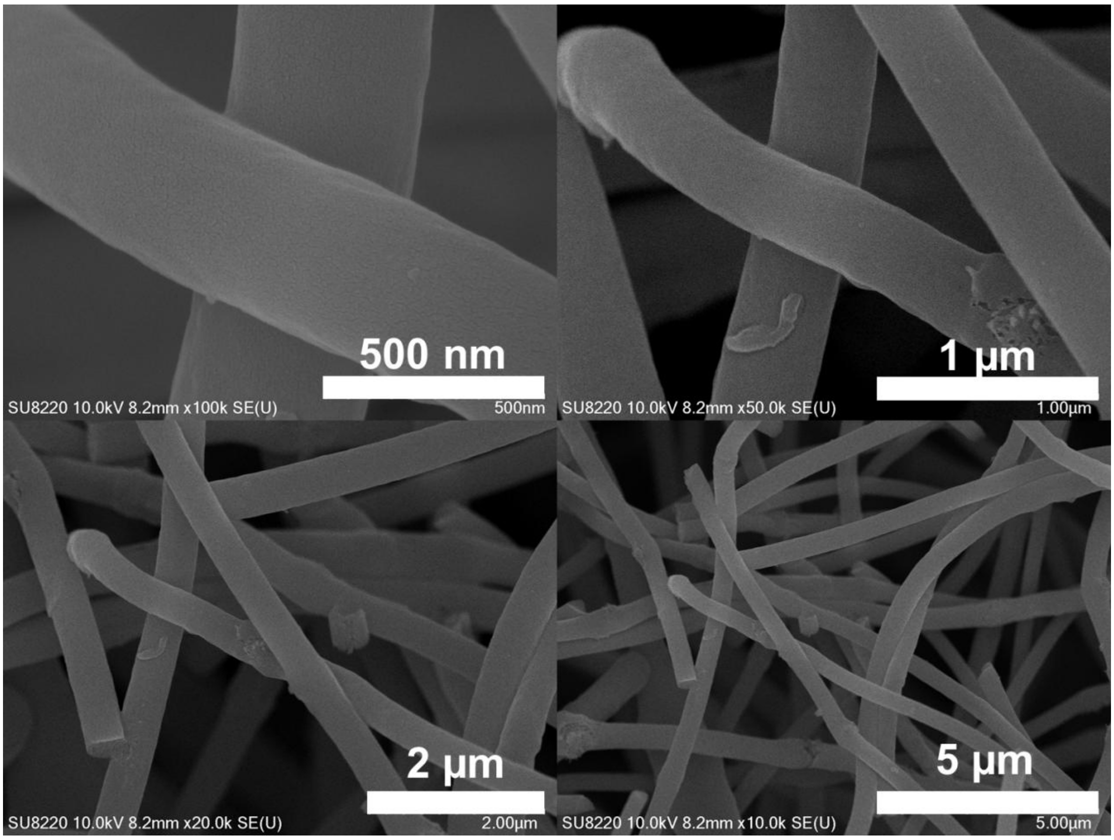

Figure S1. SEM characterization of FeMn@C/CNFs at varying scales.

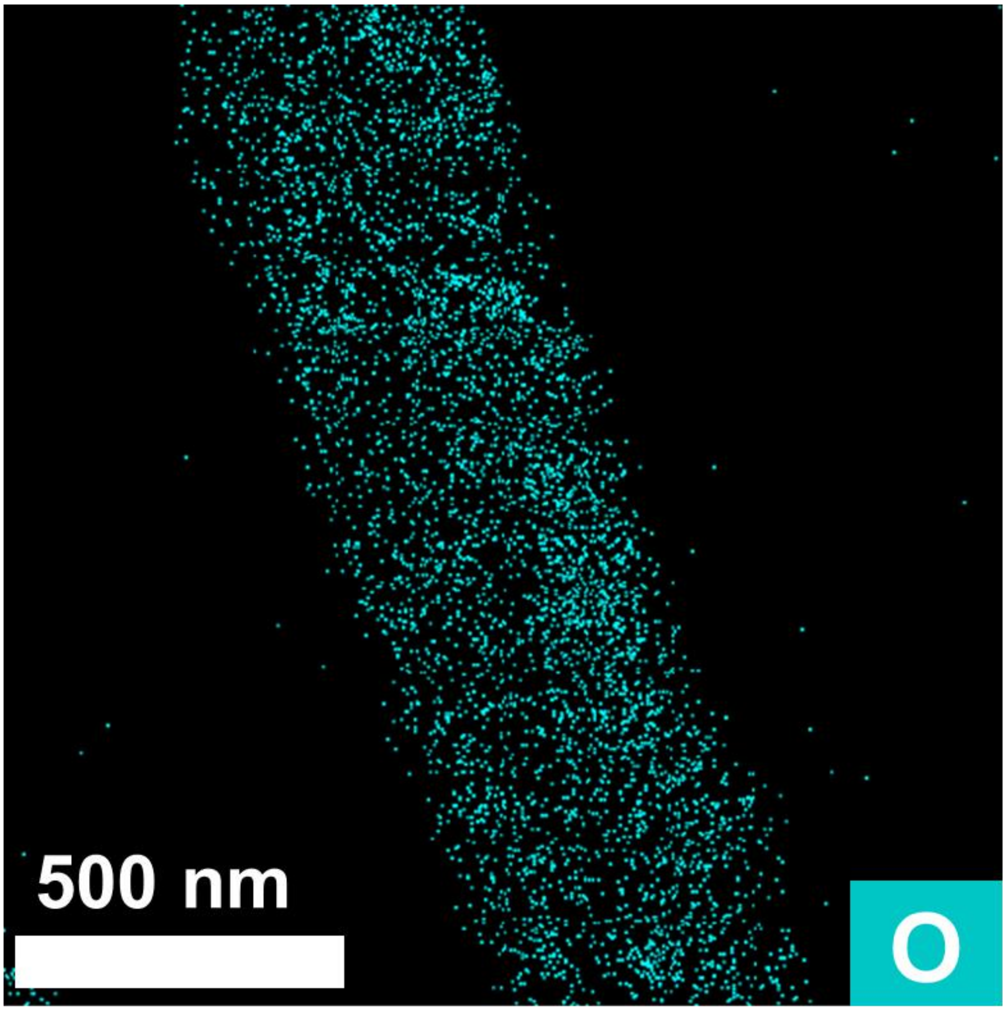

Figure S2. Mapping map of O of FeMn@C/CNFs.

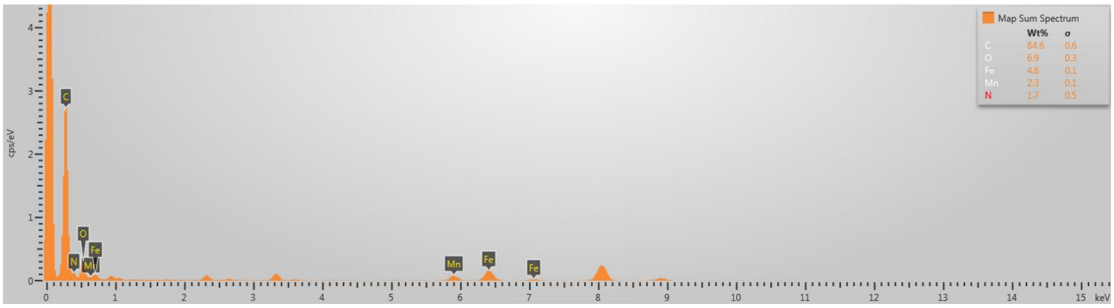

Figure S3. Characterization of the elemental content of FeMn@C/CNFs by EDS spectroscopy

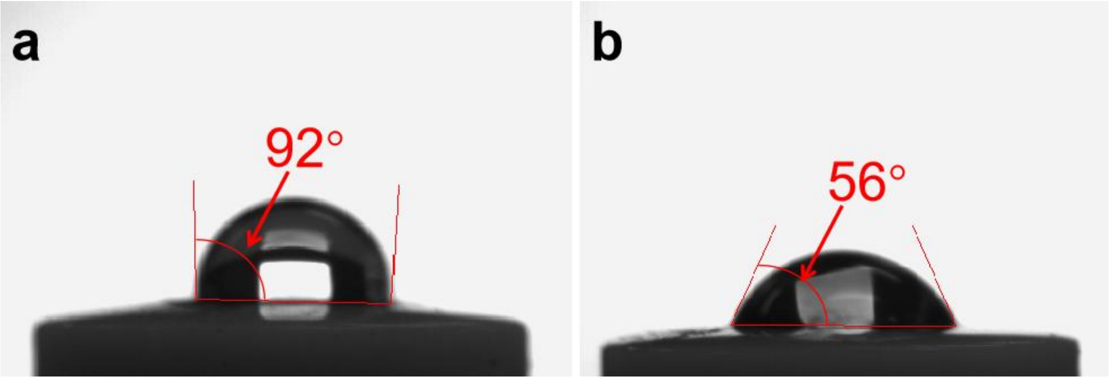

Figure S4. (a) Contact angle of bare electrode; (b) Contact angle of FeMn@C/CNFs material

Figure S4(a) shows the contact angle of the bare electrode, and Figure S4(b) shows the contact angle of the FeMn@C/CNFs material. From the figure, it can be seen that when FeMn@C/CNFs is modified on the electrode surface, the contact angle decreases significantly, and the material becomes more hydrophilic, and in this way, it will promote electron transfer, improve the current response and signal sensitivity, shorten the mass transfer time, and improve the response speed.

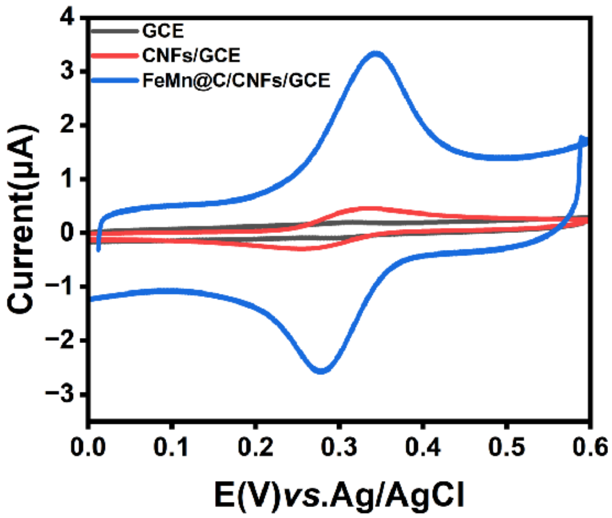

Figure S5. Comparison of CVs for different electrodes without $[Fe\ (CN)_6]^{3+/4+}$

It can be seen from the figure that when no REDOX pair $[Fe(CN)_6]^{3+/4+}$ is added, there is almost no REDOX reaction in the bare electrode. Then, when Fe and Mn are added to the modified electrode, electron transfer will be accelerated on the electrode surface. In this way, REDOX will occur even without adding the REDOX pair $[Fe(CN)_6]^{3+/4+}$.

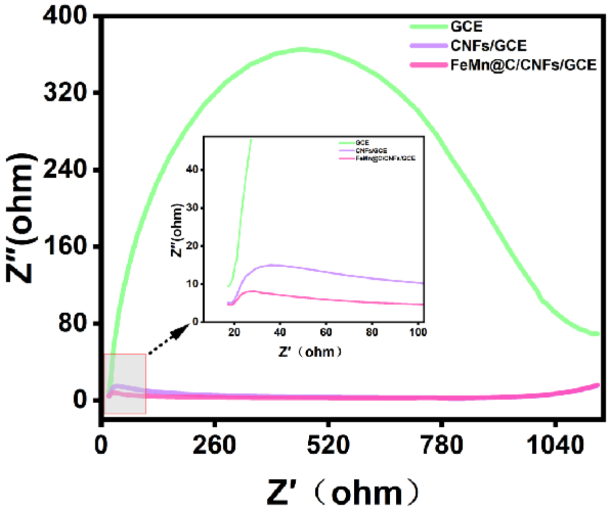

Figure S6. Impedance plots for different electrodes

The charge transfer resistanceof the modified electrode surface was characterized by electrochemical impedance spectroscopy. Generally, the lower the Rct, the higher the conductivity, and the smaller the curve radius, as shown in the figure. The impedance of the bare electrode was the highest, while FeMn@C/CNFs/GCE exhibited significantly lower impedance, indicating that its conductivity was superior to that of the bare electrode. From the figure, it can be seen that the electrochemical impedance of CNFs/GCE was between the two, indicating that the modification with CNFs would improve its electron transfer, but the effect was not as good as that of FeMn@C/CNFs/GCE, further verifying the advantages of the FeMn@C material modification.

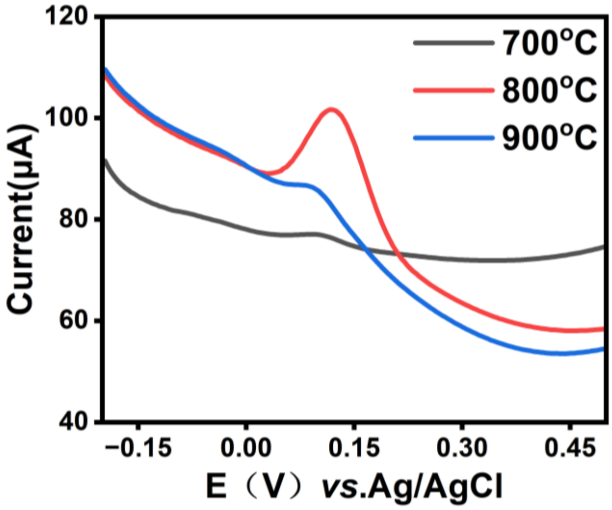

Figure S7. Comparison of materials at different calcination temperatures

To verify the influence of different calcination heating rates on the material properties, the materials were calcined at different heating rates respectively. As can be seen from Figure S7, when the annealing temperature were 700 °C and 900 °C, the signals for the target detection drugs were poor and the baselines were unstable. However, when using annealing temperature of 800°C, both the detection signal and the baseline are good.
